# DIAPH2, PTPRD and HIC1 Gene Polymorphisms and Laryngeal Cancer Risk

**DOI:** 10.3390/ijerph18147486

**Published:** 2021-07-13

**Authors:** Mirosław Śnit, Maciej Misiołek, Wojciech Ścierski, Anna Koniewska, Grażyna Stryjewska-Makuch, Sławomir Okła, Władysław Grzeszczak

**Affiliations:** 1 Department and Clinic of Internal Medicine, Diabetology and Nephrology, Medical University of Silesia, 41-800 Zabrze, Poland; msnit@sum.edu.pl; 2 Department of Otorhinolaryngology and Laryngological Oncology, Medical University of Silesia, 41-800 Zabrze, Poland; mmisiolek@gmail.com (M.M.); wojscier@mp.pl (W.Ś.); ziembicka.a@gmail.com (A.K.); 3 Department of Laryngology and Laryngological Oncology, Upper Silesian Medical Centre of Silesian Medical University, 40-055 Katowice, Poland; makuch_mg@wp.pl; 4 Department of Otolaryngology, Head and Neck Surgery, Holy-Cross Cancer Center, 25-734 Kielce, Poland; slawekok@gmail.com; 5 Department of Internal Medicine, Diabetology and Nephrology, Medical University of Silesia, ul. 3. Maja 13-15, 41-800 Zabrze, Poland

**Keywords:** DIAPH2, PTPRD and HIC1 gene polymorphisms, laryngeal cancer risk

## Abstract

AIM, DIAPH2, PTPRD and HIC1 are the cell glycoprotein, which play an important role in the occurrence and development of tumors. This study was designed to assess the association between DIAPH2, PTPRD and HIC1 SNPs and laryngeal cancer risk. PATIENTS AND METHODS: This study including 267 patients with histologically confirmed laryngeal cancer and 157 controls. The relationship between genetic variations DIAPH2 (rs6620138), PTPRD (rs3765142) and HIC1 (rs9901806) and the onset of laryngeal cancer were investigated. Statistical analysis to calculate the relationship between DIAPH2, PTPRD and HIC1 genes polymorphism and pathogenesis of laryngeal cancer. RESULTS: The results showed that rs6620138 DIAPH2 polymorphism could increase the onset risk of laryngeal cancer. Statistically significant differences in allele distribution of rs6620138 DIAPH2 and rs9901806 HIC1 in the case and control groups subgroups. CONCLUSIONS: This study results suggested that genetic variation of rs6620138 DIAPH2 polymorphism is related to the susceptibility to laryngeal cancer. Our results provide a basis to begin basic research on the role of DIAPH2 gene in the pathogenesis of laryngeal cancer.

## 1. Introduction

Laryngeal cancer is one of the most common respiratory malignancies [1]. Among respiratory cancers, the incidence of laryngeal cancer ranks it second after lung cancer [2,3]. The incidence ratio of laryngeal cancer in men to women is more than about eight to one [4]. The incidence increases in the fifth decade of life and peaks in the seventh and eighth decades [5]. Most (95%) of these cancers are squamous cell carcinomas [6]. The incidence and mortality of laryngeal cancer worldwide was up to 2.76 cases/year per 100,000 population and 1.66 deaths/year per 100,000 population, respectively [7]. Despite the continuous improvement of laryngeal cancer treatments and the continuous development of detection techniques, the overall survival rate of patients with laryngeal cancer was not significantly improved, and the survival rate for advanced laryngeal cancer was low [8].

The cause of laryngeal cancer is still not fully understood. Epidemiological studies have shown that environmental (smoking and drinking alcohol) are important risk factors for laryngeal cancer [9]. Nevertheless, only a few smokers and drinkers, living in a similar environment (same air pollution, same noise level, very comparable diet, working in similar conditions, and resting in similar places) develop and suffer from laryngeal cancer. This may indicate that although environmental factors can lead to cancer, the degrees of sensitivity of different individuals to environmental carcinogens vary. This suggests that the occurrence of laryngeal cancer is associated with the presence of genetic factors responsible for its development [10,11].

Among the genetic factors studied are the presence of genes responsible for the production of certain proteins and enzymes and their polymorphisms [12,13,14,15].

Researchers conducted a study to find the relationship between the polymorphisms of the studied genes and the risk of developing laryngeal cancer. The genes studied include: glucose transporter isoform 1 (GLUT1) reference single nucleotide polymorphism (rs) 710218, hypoxia inducible factor 1 alpha (HIF1α) rs11549465 and transcription factor T-box 21 (TBX21) protein rs17250932. Polymorphisms of GLUT1, HIF1α and TBX21 genes had no effect on laryngeal cancer development [16]. S. Ekizoglu et al. indicate that the gene SLC22A23 (solute carrier family 22, member 23) may play a role in laryngeal cancer development [17]. The telomerase reverse transcriptase gene TERT-CLPTM1L, located on chromosome 5p15.33, plays a key role in the formation and progression of various cancers. Yu J et al. reported that the TERT-CLPTM1L gene may be a significant biomarker of susceptibility to oropharyngeal and laryngeal cancers [18]. An interesting study has been done on the significance of the association between CD14 gene polymorphism and laryngeal cancer risk [19].

In our observation of genetic risk of laryngeal cancer, we considered three genes: the DIAPH2 (diaphanous related formin 2) gene, the PTPRD (protein tyrosine phosphatase receptor Type D) gene and the HCI1 (hypermethylated-in-cancer 1) gene.

The DIAPH2 (diaphanous related formin 2) gene is located on the long arm (q) of chromosome X at position 21, 23. The product of this gene belongs to the diaphanous subfamily of the formin homology protein family. This gene may play a role in the development and normal function of many organs. Alterations in DIAPH2 increase cell motility and may contribute to the metastatic potential of laryngeal squamous cell carcinoma [20]. High expression of diaphanous related formin 2 (DIAPH2) was associated with poor overall survival in head and neck squamous cell carcinoma and laryngeal squamous cell carcinoma (LSCC). DIAPH2 is upregulated in LSCC and may act as an oncogene by inhibiting apoptosis through the ATR/p53/caspase-3 pathway in LSCC cells [21].

PTPRD (protein tyrosine phosphatase receptor Type D) gene is located on chromosome 9, specifically p 24.1–p 23. PTPRD is the enzyme encoded by this gene. The encoded protein is a signaling molecule that regulates many cellular processes including cell growth, differentiation, mitotic cycle and oncogenic transformation. Association of mutations in this gene has been shown to be associated with the risk of developing malignancies, including lymphomas and various types of cancer [22]. The Cancer Genome Atlas dataset suggested that DNA methylation is the main mechanism of PTPRD silencing in these tumors. Authors provide further evidence of the high incidence of PTPRD inactivation in laryngeal squamous cell carcinoma. Deletions and loss of function mutations are responsible for PTPRD loss only in a fraction of cases, whereas DNA methylation is the dominating mechanism of PTPRD inactivation [23].

The HCI1 (hypermethylated-in-cancer 1) gene is located on chromosome 17, specifically 17p13.3, in a frequently hypermethylated region [24]. HIC1 encodes a transcriptional repressor involved in DNA damage response and in complex regulatory loops with P53 and SIRT1 [25]. This gene is frequently deleted or epigenetically silenced.in many human cancers. Hypermethylated-in-cancer 1 (HIC1) is a tumor suppressor gene frequently inactivated by epigenetic silencing and loss-of heterozygosity in a broad range of cancers. Loss of HIC1, a sequence-specific zinc finger transcriptional repressor, results in deregulation of genes that promote a malignant phenotype in a lineage-specific manner. In particular, upregulation of the HIC1 target gene SIRT1, a histone deacetylase, can promote tumor growth by inactivating TP53. Loss of HIC1 expression in the early stages of tumor formation may contribute to malignant transformation through the acquisition of chromosomal instability [24].

## 2. Aim

The aim of our study was to determine the relationship between genetic variations DIAPH2 (rs6620138), PTPRD (rs3765142) and HIC1 (rs9901806) and laryngeal cancer risk in 267 patients with histologically confirmed laryngeal cancer and 157 controls.

## 3. Patients and Methods

The protocol of this study was approved by the Ethics Committee of the Medical University of Silesia (Katowice, Poland). The population study consisted of 267 subjects with histologically confirmed laryngeal cell carcinoma, treated in the Department of Otorhinolaryngology and Laryngological Oncology, Medical University of Silesia, Zabrze, Poland, Department of Laryngology and Laryngological Oncology, Upper Silesian Medical Centre of Silesian Medical University, Katowice, Poland and Department of Otolaryngology, Head and Neck Surgery, Holycross Cancer Centre, Kielce, Poland. All the patients were clinically at stage T3 T4, N0, N3 and M0 and underwent laryngectomy. Paraffin embedded 20 µm tissue sections were collected from tumor and corresponding healthy tissues. In this group subjects DNA was isolated from material for DNA isolation was collected from each subject from his histopathological section. The material for the research was obtained in cooperation with the Chair and Department of Pathomorphology of the Silesian Medical University in Zabrze. In order to perform the necessary genetic tests, the MagCore Genomic DNA FFPE One-Step kit of the MagCore isolation system was used, this material was later used for genetic studies (allelic discrimination of three polymorphisms of laryngeal cancer genes.

The control group consisted of 157 subjects who did not have laryngeal or other cancers, of comparable age and similar gender distribution. In the control group, 5 mL of venous blood was collected from each subject using a vacuum anticoagulation tube containing EDTA and placed at −70 °C to be left to stand.

A standardized epidemiological questionnaire was developed. Demographic and environmental exposure data of each subject were examined, including sex, age, smoking, alcohol drinking, history of individual diseases and family history of cancer. Information on smoking and alcohol drinking history was declarative, taken from the medical history. For smokers, the number of years smoked was calculated.

### 3.1. SNP Selection

Based on the English references and bioinformatics database (http://www.ncbi.nlm.nih.gov/snp/) (accessed 10 January 2020), three SNP loci rs6620138, rs3765142, rs9901806, sequentially in the genes DIAPH2, PTPRD, HIC1. Using primers: DIAPH2 rs6620138 Context Sequence [VIC/FAM]. CTGGGCTCCCACTCCAGAGGGCAGC[C/T]GGTCCTTCGCCGGTGCCCAGGCCGC, PTPRD rs3765142 Context Sequence [VIC/FAM]. AATTGCATATGACTCATTTCCTCTT[A/T]GAAACCATGAGCTCCCTTCATAGGG HICI rs9901806 Context Sequence [VIC/FAM] CTGGGCTCCCACTCCAGAGGGCAGC[C/T]GGTCCTTCGCCGGTGCCCAGGCCGC

### 3.2. Genotyping

DNA was extracted from all samples using kits for DNA isolation MagCore Genomic DNA FFPE One-Step Kit and the genotype was identified via ROCHE LifeCycler 96. Real-time fluorescent quantitative PCR was done by using specific assays TaqMan genotyping method. Two blank controls (MQ) they were placed for marking each time in each 384-well detection unit as the quality control. Three genotypes of each patient were determined, and their frequency was determined.

### 3.3. Statistical Analysis

Statistical analysis system (STATISTICA) was used for data collation and statistical analysis. *p* < 0.05 suggested that the difference was statistically significant. All statistical tests were two-sided probability tests. x2 test was used for the differences of distribution frequencies in demographic characteristics, environmental exposure parameters (gender, age, smoking and drinking) and each genotype between case group and control group. Whether the genotype frequency of normal control group met the Hardy–Weinberg equilibrium law, it was calculated to confirm the randomness of selecting subjects.

## 4. Results

### 4.1. General Characteristics of Patients from Case and Control Group

A total of 257 patients with laryngeal cancer were included in the study group and 157 in the control group. The subjects did not differ from the control group in age (both in the total group and in the male and female participants) as well as in the proportion in both male and female participants (Table 1).

The basal analysis of subgroups (smokers, nonsmokers, drinkers, nondrinkers, smokers and drinkers, smokers and nondrinkers, nonsmokers and drinkers and nondrinkers and nonsmokers) are presented in Table 2. We didn’t find statistically significant differences in age of subjects and sex distribution of subjects between case and control groups.

### 4.2. Distribution of Three SNP Loci; rs6620138, rs3765142 and rs9901806, Sequentially in the Genes DIAPH2, PTPRD and HIC1 in the Case and Control Groups

The distribution frequencies of loci in the case and control groups met the Hardy–Weinberg equilibrium law (*p* > 0.05). The frequencies of above mentioned genotypes and alleles DIAPH2, PTPRD and HIC1 genes in the case group and the control group were presented in Table 3 In our study, we found a statistically significant difference between the case and control groups in the distribution of genotypes and alleles of rs6620138 DIAPH2 (chi square test—*p* < 0.001). We did not find similar significance in the distribution of genotypes and alleles of rs3765142 PTPRD and rs9901806 HIC1 between the case and control groups.

### 4.3. Genotypes Distribution of Three SNP Loci; rs6620138, rs3765142 and rs9901806, Sequentially in the Genes DIAPH2, PTPRD and HIC1 in Subgroups of the Case Group

Genotypes distribution of three SNP loci; rs6620138, rs3765142 and rs9901806, sequentially in the genes DIAPH2, PTPRD and HIC1 in subgroups of the case group were presented in Table 4. In our case group, we found a statistically significant difference between smokers and nonsmokers in the case group in the distribution of genotypes and alleles of rs6620138 DIAPH2 (chi-square test–*p* < 0.005). We did not find similar significance in the distribution of genotypes and alleles of rs3765142 PTPRD and rs9901806 HIC1 between subgroups in the case (Table 4).

### 4.4. Genotypes Distribution of Three SNP Loci; rs6620138, rs3765142 and rs9901806, Sequentially in the Genes DIAPH2, PTPRD and HIC1 in the Control Group

Genotypes distribution of three SNP loci; rs6620138, rs3765142 and rs9901806, sequentially in the genes DIAPH2, PTPRD and HIC1 in subgroups of the control group were presented in Table 5. We did not find statistically significance in the distribution of genotypes of rs6620138 DIAPH2, rs3765142 PTPRD and rs9901806 HIC1 between subgroups in the control group (Table 5). We found significance in the distribution of alleles of rs6620138 DIAPH2, rs3765142 PTPRD and rs9901806 HIC1 between subgroups in the control group. See Table 5b for details.

### 4.5. Comparison of Alleles Distribution between Subgroups of the Case and Control Groups

Alleles distribution of three SNP loci; rs6620138, rs3765142, rs9901806 sequentially in the genes DIAPH2, PTPRD, HIC1 between subgroups of the case and control groups were presented in Table 6. We found statistically significant differences in allele distribution of rs6620138 DIAPH2 and rs9901806 HIC1 in the case and control groups subgroups. See Table 6 for details.

## 5. Discussion

Laryngeal cancer is one of the most common malignant neoplasms of the respiratory system [1,2]. In the etiopathogenesis of laryngeal cancer, in addition to environmental factors such as smoking, drinking alcohol and air pollution, genetic factors also play an important role [9,10,11].

The aim of our study was to try to find a link between the studied polymorphisms of the DIAPH2, HIC1 and PTPRD genes and the risk of laryngeal cancer development.

We enrolled another 267 patients, Caucasian, with histopathologically confirmed cancer of the larynx and 157 people, Caucasian who did not suffer from it. The subjects were not significantly different in age or sex distribution from the control group (Table 1). The control subjects had similar exposure to environmental factors (similar amount of smoking, drinking alcohol, living in an environment with similar pollution and in the same climate zone) (Table 1 and Table 2). We hypothesized that if we showed a difference in the distribution of the polymorphisms of the DIAPH2, HIC1 and PTPRD genes studied, this would indicate a significant effect of these genetic variations on the risk of developing laryngeal cancer.

In our study, we first compared the distribution of the studied genotypes and alleles between the two whole groups of subjects (case and control). We found no differences in the distribution of genotypes and alleles of the studied HIC1 and PTPRD polymorphisms between the case and control groups (Table 3). However, we found a significant difference in the distribution and alleles of DIAPH2 (*p* < 0.001) between the case and control group (Table 3). In the control group, the frequency of the A rs6620138 allele of the DIAPH2 gene was significantly higher than in the study group. In this situation, it can be suggested that people with the allele described above have a lower risk of developing laryngeal cancer than those without this allele. In order to confirm this suggestion, it would be necessary, firstly, to carry out observations in a larger study group and in a control group; secondly, it would be necessary to answer the question whether the relationship is similar in individuals of other races. Finally, the A/T allele distribution of the rs6620138 polymorphism of the DIAPH2 gene is only 0.2/0.8. However, if the obtained results were confirmed by other authors, the determination of the allele of the mentioned gene could have a practical meaning in predicting the risk of developing laryngeal cancer.

In the second part of the study, we observed whether in patients with laryngeal cancer there were differences in the distribution of genotypes and alleles of DIAPH2, HIC1 and PTPRD polymorphisms between smokers and nonsmokers, between drinkers and nondrinkers, between smokers and drinkers and nonsmokers and nondrinkers and between smokers and nonsmokers. The results are presented in Table 4. Consequently, however, we found only a significant difference in the distribution of rs6620138 DIAPH2 between subgroups smokers and nonsmokers in the case group, HIC1 and PTPRD genotypes and alleles between the study subgroup patients with laryngeal cancer (Table 4). The results of the study suggest that after subgrouping, the subgroups were too small to obtain a possible statistical relationship. It follows that the study group must include a larger number of subjects.

We conducted a similar observation in healthy people. We examined whether there were differences in the distribution of genotypes and alleles of DIAPH2, HIC1 and PTPRD gene polymorphisms between smokers and nonsmokers, between drinkers and nondrinkers, between smokers and drinkers and between nonsmokers and nonsmokers, and between smokers and nonsmokers and nonsmokers and drinkers in the control group. Consequently, however, showed a significant difference in the distribution of DIAPH2, HIC1 and PTPRD alleles between the subgroups in the control group. The results are presented in Table 5. The results obtained in the control group are very interesting. They suggest that environmental influences may play a greater role than the genetic factors studied. This calls for further observations in this regard.

In the final part of the study, we compared smokers with smokers, nonsmokers with nonsmokers, drinkers with drinkers, nonsmokers with nonsmokers, drinkers and smokers with drinkers and smokers, smokers and nonsmokers with smokers and nonsmokers and nonsmokers and nonsmokers with nonsmokers in the study group and control group. The results are presented in Table 6. Consequently, however, we didn’t show a significant difference in the distribution of PTPRD genotype between the study subgroup patients with laryngeal cancer and control subgroups. We show a significant difference in the allele’s distribution of DIAPH2 and HIC1 between the study subgroup patients with laryngeal cancer and control subgroups (Table 6). This suggests, on the one hand, that the significance of the gene polymorphisms DIAPIH2 and HIC1 studied is different in patients with laryngeal cancer and in healthy subjects, and, on the other hand, that in order to obtain more reliable, unambiguous results of the study, both groups should be significantly enlarged.

## 6. Conclusions

The relationship between the genetic variation of DIAPH2, PTPRD and HIC1 genes and the genetic susceptibility to laryngeal cancer was investigated in this study for the first time.

The research showed that polymorphism rs6620138 DIAPH2 gene was associated with the pathogenesis of laryngeal cancer.

The functional mechanism of this polymorphic locus was not explored.

Statistically significant differences in allele distribution of rs6620138 DIAPH2 and rs9901806 HIC1 in the case and control groups subgroups was observed.

The sample size in this study was relatively smaller, so large-sample experimental studies are further needed.

## Figures and Tables

**Table 1:** Selected characteristics in laryngeal cancer cases and controls.

Varables	CASE—*N* (%)	CONTROL—*N* (%)	*p*<
All subjects	267 (100%)	157 (100% )	
Age			
All	62.0 ± 8.5	61.6 ± 4.9	NS
Women	63.5 ± 8.1	60.9 ± 4.2	NS
Men	61.6 ± 8.3	61.8 ± 3.9	NS
Gender			NS
Women	31 (12%)	26 (16%)	
Men	236 (88%)	131 (84%)	

No statistically significant differences in age of subjects and sex distribution of subjects between case and control groups.

**Table 2:** Age and sex distribution in laryngeal cancer case group and control group.

Case/Control	Smokers	Non-Smokers	Drinkers	Nondrinkers	Smokers and Drinkers	Smokers and Nondrinkers	Nonsmokers and Drinkers	Nonsmokers and Nondrinkers
Case *N* (%)	227 (85)	40 (15)	206 (77)	61 (23)	180 (67)	46 (7)	26 (10)	15 (6)
Mean age	63.0 ± 8.5	65.5 ± 7.4	62.3 ± 8.4	65.5 ± 8.4	65.1 ± 3.6	65.0 ± 8.4	62.4 ± 8.2	65.1 ± 3.6
Men/Women	202/25	34/6	185/21	50/11	162/18	39/7	23/3	12/3
Men age	62.6 ± 8.8	65.5 ± 7.4	62.6 ± 8.4	64.5 ± 8.4	64.8 ± 3.6	63.4 ± 8.4	64.4 ± 8.2	65.0 ± 3.6
Women age	63.6 ± 8.8	63.3 ± 4.9	62.1 ± 8.7	66.6 ± 8.7	65.4 ± 4.1	66.6 ± 6.4	60.0 ± 1.7	65.5 ± 3.0
Control *N* (%)	120 (76)	37 (24)	94 (60)	63 (40)	66 (42)	54 (34)	28 (18)	9 (6)
Mean age	62.9 ± 4.0	64.2 ± 7.1	61.5 ± 3.9	61.5 ± 4.1	61.8 ± 4.1	61.7 ± 3.9	61.5 ± 3.9	63.4 ± 5.5
Men/Women	105/15	26/11	80/14	51/12	54/12	51/3	28/0	9/0
Men age	62.0 ± 4.0	64.9 ± 7.9	61.6 ± 7.4	62.0 ± 3.9	61.9 ± 4.7	62.0 ± 3.9	62.4 ± 4.7	63.4 ± 5.5
Women age	60.2 ± 3.5	63.3 ± 4.9	61.3 ± 3.4	60.3 ± 5.0	61.1 ± 3.4	57.0 ± 3.8	61.0 ± 3.3	0

No statistically significant differences in age of subjects and sex distribution of subjects between case and control groups.

**Table 3:** Genotypes distribution in laryngeal cancer case group and control group. alleles distribution in laryngeal cancer case group and control group.

**(a) DIAPH2 PTPRD HICI.**									
	**AT**	**AA**	**TT**	**TT**	**AT**	**AA**	**CT**	**TT**	**CC**
Case	23–8.6%	49–18.4%	195–73.0%	133–49.8%	103–38.6%	31–11.6%	123–46.1%	107–40.1%	37–13.8%
Control	48–30.6%	17–10.8%	92–58.6%	79–50.3%	65–41.4%	13–8.3%	63–40.1%	71–45.2%	23–14.6%
**(b) DIAPH2 PTPRD HICI.**									
	**A**		**T**	**T**	**A**		**C**	**T**	
Case	101–18.9%		413–81.1%	369–69.1%	165–30.9%		197–36.9%	337–3.11%	
Control	82–26.1%		184–73.9%	223–71.0 %	81–29.0%		109–34.7 %	205–65.3%	

(a) significant difference in rs6620138 DIAPH2 distribution between case and control (chi-square test—*p* < 0.001). (b) significance difference in DIAPH2 (rs6620138) distribution between case and control (chi-square test—*p* < 0.001), no other.

**Table 4:** Genotypes and alleles distribution in the case group.

**(a) DIAPH2 PTPRD HIC1.**									
**CASE**	**AT**	**AA**	**TT**	**TT**	**AT**	**AA**	**CT**	**TT**	**CC**
Smokers	21 – 9,3%	39–17.3%	166–73.4%	114–50.4%	83–36.7%	29–12.9%	104–46.0%	90–39.8%	32–14.2%
Nonsmokers	2–4.9%	10–244.4%	29–70.7%	19–46.3%	20–48.8%	2–4.9%	19–46.3%	17–41.5%	5–12.2%
Drinkers	22–10.7%	36–17.6%	147–71.7%	100–48.8%	78–38.0%	27–13.2%	94–45.8%	85–41.5%	26–12.7%
Nondrinkers	1–1.6%	13–21.3%	47–77.1%	33–54.1%	24–38.3%	4–6.6%	28–45.9%	22–36.1%	11–18.0%
Smokers/Nondrinkers	1–2.2%	8–17.3%	37–80.5%	23–50.0%	19–41.3%	4–8.7%	23–50.0%	15–32.6%	8–17.4%
Nonsmokers/Drinkers	2–7.7%	5–19.2%	19–73.1%	9–34.6%	15–57.7%	2–7.7%	14–53.8%	10–38.5%	2–7.7%
Smokers/Drinkers	20–11.1%	31–17.2%	129–71.7%	91–50.5%	64–35.5%	25–13.0%	81–45.0%	75–41.7%	24–53.3%
Nonsmokers/Nondrinkers	14–28.6%	1–2.0%	34–69.4%	18–36.7%	26–53.1%	5–10.2%	19–38.8%	18–36.7%	12–24.5%
**(b) DIAPH2 PTPRD HIC1.**									
**CASE**	**A**	**T**	**ALLELE**	**T**	**A**	**ALLELE**	**C**	**T**	**ALLELE**
Smokers	99	353	452-A (0.22:0.78)	311	141	452-A (0.23:0.77)	168	284	452-C (0.36:0.64)
Nonsmokers	22	60	82-A (0.27:0.73)	58	24	82-A (0.29:0.71)	29	53	82-C (0.35:0.65)
Drinkers	94	316	410-A (0.23:0.77)	278	132	410-A (0.32:0.68)	146	264	410-C (0.36:0.64)
Nondrinkers	27	95	122-A (0.22:0.78)	90	32	122-A (0.26:0.74)	50	72	122-C (0.41:0.59)
Smokers/Nondrinkers	17	75	92-A (0.18:0.82)	65	27	92-A (0.29:0.71)	39	53	92-C (0.42:0.58)
Nonsmokers/Drinkers	12	40	52-A (0.23:0.77)	33	19	52-A (0.37:0.63)	18	34	52-C (0.35:0.65)
Smokers/Drinkers	82	278	360-A (0.23:0.77)	246	114	360-A (0.32:0.68)	129	231	360-C (0.36:0,64)
Nonsmokers/Nondrinkers	16	82	98-A (0.16:0.84)	62	36	98-A (0.37:0.63)	43	55	98-C (0.44:0.56)

(a) no statistically significant differences in genotypes distribution of subjects between subgroups of cases. (b) significance difference in DIAPH2 (rs6620138) alleles distribution between subgroups smokers and nonsmokers in the case group (chi- square test—*p* < 0.005), no other.

**Table 5:** Genotypes and alleles distribution in the control group. Comparison of alleles distribution in the control group.

**(a)**										
**DIAPH2 PTPRD HICI.**										
**Control**	**AT**	**AA**	**TT**	**TT**		**AT**	**AA**	**CT**	**TT**	**CC**
Smokers	32–30.5%	16–15.2%	57–54.3%	60–57.2%		37–35.2%	8–7.6%	42–40%	53–50.5%	10–9.5%
Nonsmokers	16–30.8%	1–1.9%	35–66.3%	19–36.5%		28–53.9%	5–9.6%	21–40.4%	18–34.6%	13–25.0%
Drinkers	21–28.0%	4–5.3%	50–66.3%	34–45.3%		34–45.3%	7–9.4%	32–42.7%	27–36.0%	16–21.3%
Nondrinkers	27–32.9%	13–15.9%	42–51.2%	45–54.9%		31–37.8%	7–9.4%	31–37.8%	44–53.6%	7–8.5%
Smokers/Nondrinkers	25–31.6%	13–16.5%	41–51.9%	44–55.7%		29–36.7%	6–7.6%	29–36.7%	44–55.7%	6–9.6%
Nonsmokers/Drinkers	14–28.6%	1–2.0%	34–69.4%	18–36.7%		26–53.1%	5–10.2%	19–38.8%	18–36.7%	12–24.5%
Smokers/Drinkers	7–26.9%	3–11.5%	16–61.5%	16–61.5%		8–30.8%	2–7.7%	13–50%	9–34.6%	4–15.4%
Nonsmokers/Nondrinkers	2–66.7%	0%	1–33.3%	1–33.3%		2–66.7%	0%	2–66.7%	0%	1–33.3%
**DIAPH2 PTPRD HICI.**										
**Control**	**A**	**T**	**ALLELE**	**T**	**A**	**ALLELE**		**C**	**T**	**ALLELE**
Smokers	66	146	212-A (0.31:0.69)	157	53	212-A (0.25:0.75)		62	148	212-C (0.29:0.71)
Nonsmokers	18	86	104-A (0.17:0.83)	66	38	104-A (0.37:0.63)		47	57	104-C (0.20:0.80)
Drinkers	29	121	150-A (0.24:0.76)	102	48	150-A (0.32:0.68)		64	86	150-C (0.43:0.57)
Nondrinkers	53	111	164-A (0.32:0.68)	104	60	164-A (0.37:0.63)		45	119	164-C (0.27:0.73)
Smokers/Nondrinkers	51	107	158-A (0.32:0.68)	117	41	158-A (0.26:0.74)		41	117	158-C (0.26:0.74)
Nonsmokers/Drinkers	16	82	98-A (0.16:0.84)	62	36	98-A (0.37:0.63)		43	55	98-C (0.44:0.56)
Smokers/Drinkers	13	39	52-A (0.25:0.75)	40	12	52-A (0.23:0.77)		21	31	52-C (0.40:0.60
Nonsmokers/Nondrinkers	2	4	6-A (0.33:0.67)	4	2	6-A (0.33:0.67)		4	2	6-C (0.67:0.33)
**(b)**										
**Control**			**DIAPH2**			**PTPRD**			**H1CI**	
Smokers/Nonsmokers			0.018			0.038			0.0000	
Drinkers/Nondrinkers			0.012			NS			0.0001	
Smokers/Nondrinkers-Nonsmokers/Drinkers			NS			NS			0.003	
Smokers/Drinkers-Nonsmokers/Nondrinkers			NS			NS			NS	

Non statistically significant differences between examined subgroups in the control group.

**Table 6:** Comparison of alleles distribution between the case and the control groups.

	DIAPH2	PTPRD	H1CI
Smokers	0.0000	NS	0.0000
Nonsmokers	NS	NS	NS
Drinkers	NS	NS	0.0000
Nondrinkers	NS	NS	0.0000
Smokers/Nondrinkers	NS	NS	0.000
Nonsmokers/Drinkers	0.024	NS	0.0121
Smokers/Drinkers	NS	NS	0.001
Nonsmokers/Nondrinkers	NS	NS	NS

## Data Availability

No applicable.
